# What must come down goes up - the effect of noise on weights in spike-timing-dependent plasticity

**DOI:** 10.1186/1471-2202-16-S1-P283

**Published:** 2015-12-18

**Authors:** Michael Klein, Angelo Cangelosi, Thomas Wennekers

**Affiliations:** 1 Centre of Robotics and Neural Systems, Plymouth University, PL4 8AA, Plymouth, UK

## 

**Figure 1:**
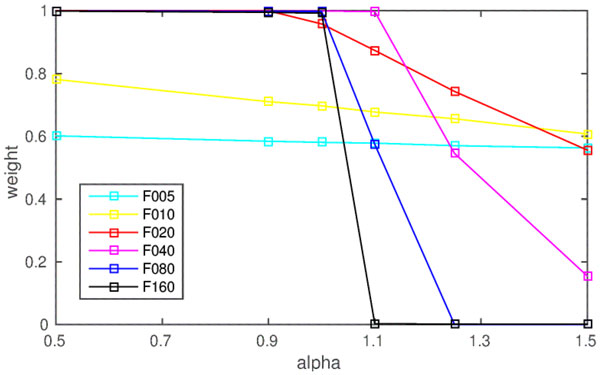
A plot of the weight strength after training for different frequencies with an initial weight of 0.5. As expected, the weight increases for α-parameters smaller than 1. However, the weight also increases for α-parameters larger than 1 for frequencies below 80Hz.

In large-scale spiking neural models that learn with spike-timing-dependent plasticity (STDP), it is a crucial, but difficult problem to balance synaptic potentiation (necessary to learn a task) and synaptic depression (necessary to counteract accidental weight increase and to balance overall firing rates). Adding random noise and choosing parameters such that inhibition slightly dominates excitation [1,2] is considered one way of accomplishing this. With the learning rule being standard STDP [2], the parameter that determines whether inhibition or excitation dominates is the so-called α-parameter, defined as the ratio between parameters that determine amount of depression and those that determine amount of potentiation [3]. If the α-parameter is set to a value greater than 1, the weights are assumed to go down. However, using numerical simulations, we demonstrate that this is not the case. Using standard leaky integrate-and-fire neurons, we ran 10 simulations per parameter set (250s each), with α-parameters A = {0.5, 0.9, 1.0, 1.1, 1.25, 1.5} and noise frequencies F = {5, 10, 20, 40, 80, 160}. We also varied the maximum weight and the initial weight of the synapse from 0 to 1 in small intervals. In contrast to our assumptions, we find a weight increase with α-parameters greater than 1 (see Figure 1). The complex weight dynamics observed can be explained by the interplay of 3 factors. 1. The α-parameter: if it is greater than 1 it tends to drive the weights down, given that firing is uncorrelated. 2. The impact of the pre-synaptic spike on the post-synaptic potential (determined by synaptic weight, capacitance of the neuron, or time constant of the synapse): if the impact is strong enough pre-synaptic spikes will cause post-synaptic spikes and firing will no longer be uncorrelated. Hence, weights will tend to go up (for example, if the initial values of the weights are too high). 3. Noise frequency: There is a positive interaction between noise frequency and α-parameter in driving weights down. If the frequency is high enough, the α-parameter can be low. If the frequency is low the α-parameter needs to be very high. High frequency drives the weights down, because the post-synaptic neuron operates in input averaging mode, firing more regular than the pre-synaptic spikes that evoked it, and, therefore, being decorrelated from it [2]. These findings give insights in how to set parameters (in particular α-parameter, initial weights and noise frequency) to achieve a desired weight dynamics in large-scale STDP models.
